# Eco-friendly cellulose hydrogels as controlled release fertilizer for enhanced growth and yield of upland rice

**DOI:** 10.1038/s41598-023-47922-y

**Published:** 2023-11-22

**Authors:** Dayang Fazirah Binti Abg Ahmad, Mohd Effendi Wasli, Cindy Soo Yun Tan, Zaki Musa, Suk-Fun Chin

**Affiliations:** 1 Faculty of Resource Science and Technology, Universiti Malaysia Sarawak, 94300 Kota Samarahan, Sarawak Malaysia; 2 Faculty of Applied Science, Universiti Teknologi MARA, 94300 Kota Samarahan, Sarawak Malaysia; 3 Malaysian Agricultural Research and Development Institute (MARDI), Jalan Santubong, Petra Jaya, 93050 Kuching, Sarawak Malaysia

**Keywords:** Physiology, Materials science

## Abstract

The effect of urea-loaded cellulose hydrogel, a controlled-release fertilizer (CRF) on growth and yield of upland rice were investigated in upland rice. As with the initial research, nitrogen (N) treatments were applied as CRF treatments; T2H (30 kg N ha^−1^), T3H (60 kg N ha^−1^), T4H (90 kg N ha^−1^), T5H (120 kg N ha^−1^) and recommended dose of fertilizer (RDF) at 120 kg N ha^−1^ RDF (T6U) in split application and T1 (0 N) as control. Results from this study indicated that applying CRF at the optimum N rate, T4H resulted in maximum grain yield, increasing by 71%. The analysis of yield components revealed that higher grain yield in T4H CRF was associated with an increase in panicle number and number of grains per panicle. Maximum grain N uptake of 0.25 g kg^−1^ was also observed in T4H CRF. In addition, T4H CRF recorded the highest harvest index (HI) and N harvest index (NHI) of 45.5% and 67.9%, respectively. Application of T4H CRF also recorded the highest N use efficiency (NUE) and N agronomic efficiency (NAE), 52.6% and 12.8 kg kg^−1^, respectively. Observations show that CRF with only 75% N applied (T4H) in soil improved grain yield when compared to CRF with 100% N and 100% RDF in farmers’ conventional split application. This suggested that CRF with a moderate N application might produce the highest potential yield and improved N efficiencies while enhancing crop production and further increase in N supply did not increase yield and N efficiencies. The results suggest that the application of T4H CRF for upland rice would enhance HI, N efficiencies and improve the yield of upland rice. Also, all growth parameters and yield were positively influenced by the application of CRF as a basal dose compared to split application of conventional urea fertilizers.

## Introduction

Rice (*Oryza sativa* L.) is one of the basic staple foods for more than 3 billion people worldwide. Over two billion people in Asia consume rice and rice-derived products for 60–70% of their energy needs^1, 2^. Rice is a staple food for Malaysians of all socioeconomic background. In Malaysia, rice is the main food crop which occupied an area of 685,548 hectares with a production of approximately 2.57 million tons in 2017 but decreased to 2.34 million tons in 2020^3, 4^. The self-sufficiency level in Malaysia for rice hovered around 70% from 2008 to 2017^5^. In line with the National Agricultural Policy, the government wishes to increase the rice self-sufficiency level up to 75% by 2025 to ensure food security for people^5^. Meanwhile, in Sarawak, the rice self-sufficiency level was only 38%, while the rest was imported from other countries^6^. The rice self-sufficiency level is significantly low that the Sarawak government has targeted increasing the rice self-sufficiency to 60% by 2030^6, 7^.

The increase of rice production can be achieved by efficient and good agricultural practices, water^8^ and fertilizer inputs^9, 10^. However, poor management of plant nutrients and ignorance of the Liebig’s Law of Minimum serves as a hurdle in increasing rice production^11^. Most farmers apply conventional fertilizers; it was found that the efficiency of plant uptake of the conventional fertilizers applied is below 50%^12^. Some of the contributory factors that lead to nutrient deficiencies found in crops are due to losses through heavy leaching, surface run-off, reaction with organic compounds in the soils, vaporization and volatilization^13–15^. Better fertilizer management is required as it is known that long-term cropping will alter the availability of soil nutrients^16, 17^. A promising strategy to overcome these challenges is the encapsulation of fertilizers in superabsorbent hydrogels as controlled-release fertilizers using cellulose derived from waste papers^18^. This type of fertilizer which is also known as an environmentally smart nitrogen^19^, is used to minimize the fertilizer loss from rain or irrigation water, supply nutrients for a longer period, and enhance fertilizers utilization efficiency^18^.

Therefore, the current study was designed to cover the essential aspects of urea-loaded hydrogels on the growth of rice and determine their efficacy in enhancing the fertilizer use efficiency in rice. This study aims to compare the agronomic effects of urea-loaded hydrogels and conventional urea by farmers’ fertilizers practices on the growth parameters of rice, yield and yield-attributing factors of rice and on N uptake and efficiencies.

## Experimental

### Urea-loaded hydrogel preparation

A series of cellulose-based hydrogels were fabricated by crosslinking cellulose fibers (1.0–5.0%w/v) extracted from waste papers and carboxymethylcellulose (CMC) (1.75–5.0%w/v) with epichlorohydrin (ECH) (5 mL). Hydrogels formed were oven-dried (50–60 °C) to constant weight and immersed into 2.5% (w/v) urea solution for 48 h and placed in a controlled environment. The swollen urea-loaded cellulose hydrogels were oven-dried (50–60 °C) to constant weight^18^.

### Materials

*Maswangi* (MRQ74) Mardi rice variety was used in this study. The MRQ74 rice seeds were provided by Malaysian Agricultural Research and Development Institute (MARDI), which permits the use of these rice seeds in this study, including rice cultivation and collection of seeds and vegetative parts of the rice plants for analysis, reports, publications and other purposes, as well as agricultural exposition and promotion to local society. The experimental research is comply with relevant institutional, national, and international guidelines and legislation.

### Experimental details

A pot experiment was conducted during the year of 2022 (June–December). The experimental site was established in a mini greenhouse located (1°28′09.3″N, 110°25′41.3″E) at Pusat Penyelidikan Tumbuhan (PPT), Universiti Malaysia Sarawak (UNIMAS), Kota Samarahan, Sarawak. The climate was tropical rainforest characterized by its high temperature (31–33 °C) and rainfall (which may vary from nearly nothing to 700 mm or more in a day) during the entire rice growth period.

Topsoil (0–30 cm depth) was collected at the same location, air-dried, ground and sieved through a 2.0 mm mesh for soil analysis^20^. Soil pH and available total N in topsoil of experimental site were quantified^21, 22^. Total N in topsoil was 0.141%, with 0.6% P, 0.83% K, 1.5 mg/Kg Zn, 23.7 mg/Kg Fe, 2.7 mg/Kg Mn, and 1.36 mg/Kg Cu^13^. Soil pH was 4.83 and measured using Sonkir Soil pH meter MS02.

Planting pots with an inside top diameter of 30.5 cm, 26.6 cm bottom diameter and 30 cm in height were used. Each pot was filled with 12 kg of homogenous topsoil. The experiment comprised 6 treatments (Table 1) with 5 replications for each treatment to account for variation between rice plants in completely randomized block design. Each treatment was arranged in a separate block of pots at different coordinates in the greenhouse. The experiment was conducted with different treatments to minimize the effect of variation from the experiment occurring at different times. Treatments also consisted of the recommended dose of fertilizers (RDF) according to the nutrient requirement for upland rice cultivation in previous studies^15, 20, 23, 24^. Hence, a series of RDF was selected and as a control, T1 with no fertilizer applied, 30 kg N ha^−1^ in T2H (25% RDF), 60 kg N ha^−1^ in T3H (50% RDF), 90 kg N ha^−1^ in T4H (75% RDF) and 120 kg N ha^−1^ in T5H (100% RDF). RDF for 12 kg of soil in pots was calculated using Eq. (1)^25^ and were applied as in Table 1 as an initial study. Urea (46% N) was used as the source of fertilizer.

**Table 1:** List of treatments with different N fertilizers doses.

Treatments	Amount of N fertilizers
T1	0 N (control)
T2H	1.6 g N pot^−1^ hydrogels = 25% RDF
T3H	3.2 g N pot^−1^ hydrogels = 50% RDF
T4H	4.8 g N pot^−1^ hydrogels = 75% RDF
T5H	6.4 g N pot^−1^ hydrogels = 100% RDF
T6U	6.4 g N pot^−1^ (100% RDF) applied with three split applications at 15, 35 and 55 DAS (days after sowing)

Paddy seeds were soaked in water to remove hollow seeds floating on the surface of water, while the filled seeds were collected and dried. After that, 6 filled seeds were introduced to a depth of 5 cm soil depth in the pots. For T2H, T3H, T4H and T5H, before rice sowing, dried urea-loaded cellulose hydrogels were placed in a pail of topsoil (8–10 cm soil depth). The urea-loaded cellulose hydrogels were covered with topsoil, and the soils in the pots were hydrated with water. The growth experiment of paddy plants lasted for 130 days. Throughout the study, no standing water was observed during the whole growing period to simulate the actual field conditions. Irrigation water was applied 10 days after sowing and with interval of 2 days water irrigation to prevent water stress on the rice plants and allow them to reach the maturity stage^26, 27^. Manual weeding and insect, pest and disease management practices were conducted in this study to minimize yield losses.

1 $${\text{Amount of fertilizer per pot }} = \, \left( {{\text{Recommended dose of fertilizer }}/{\text{ 1 hectare}}} \right) \, \times {\text{ weight of soil in a pot}}$$

### Plant growth parameters and yield

#### Biometric observation

Growth performance data were collected as plant heights (cm), number of tillers and SPAD meter readings. Data were recorded at 15, 35, 55, 75, 90, 115 and 130 days after sowing (DAS). Culm height and panicle numbers were recorded at 90 DAS. For seedling or young plants, the height was measured from the ground level to the tip of the tallest leaf. For mature plants, it is the distance from the ground level to the tip of the tallest panicle. Culm height was measured from the ground to the node of the tallest panicle. Panicle length at harvest was measured from the difference of the plant and culm height. The number of tillers was counted manually until harvesting time and tillers with at least one visible leaf were included. The number of unproductive tillers (if any) and the number of panicles at maturity (before harvest) were also recorded. The chlorophyll levels in leaves were estimated using the SPAD meter (Minolta SPAD-502DL model, Minolta, Japan), a probe that provides a rapid and non-destructive approach which enables in situ measurement^28^. The SPAD readings were taken at the middle of each leaf and averaged. All growth data were recorded in accordance with the treatments, replicates and measurement times.

#### Yield and yield component data

At maturity, panicles from each plant within each sampling were collected, placed into different bags and labelled accordingly for the determination of yield components. The filled and hollow grains were separated accordingly and counted. Total filled grains and1000-grains weight and its moisture content were determined. Grain yields for each treatment was adjusted to a standard moisture content of 14% of fresh weight. The harvested samples were oven-dried at 70 °C for three to five days and recorded as a total biomass. Percentage of filled spikelet (%) was calculated as the number of filled spikelet per panicle by a factor of 100 over the total number of spikelets per panicle^29^. The grain harvest index (HI) was calculated using the following equation^29^:

2 $${\text{HI }} = \, \left[ {{\text{Grain yield }}/ \, \left( {{\text{Grain yield }} + {\text{ Biomass}}} \right)} \right] \times {1}00$$

#### Analyses of plant samples for N uptake and nitrogen use efficiency (NUE) estimation

The N content of plant materials was determined by the modified Kjeldahl acid-digestion method using concentrated sulfuric acid^20, 25, 30^. Each plant from each sampling pots was cut at the ground at harvest after final growth performance data were collected and the panicles were harvested. Leaves were separated from the stem and placed in different bags according to the treatments. Fresh weights of the leaves and stems were recorded prior to drying. All samples were oven-dried at 70 °C for three to five days to a constant weight. Each sample was weighed and grounded using a mechanical grinder before the subsamples were taken for N determination. The N content in the vegetative parts and grains was measured by the Kjeldahl method. Total N uptake in grain and straw was calculated by multiplying the N concentration (%) in grain and straw by their respective yields^31^. N harvest index (NHI), N use efficiency (NUE) and N agronomic efficiency (NAE) were calculated using the following equations^32^.

3 $${\text{NHI }} = {\text{ N uptake in grain }}/{\text{ N uptake in plant}}$$

4 $${\text{NUE }} = \, [{\text{N uptake }}\left( {{\text{added}}} \right) - {\text{N uptake }}\left( {{\text{control}}} \right)] \, /{\text{ Total N applied}} \times {1}00$$

5 $${\text{NAE }} = \, ({\text{Yn}} - {\text{Yo}}) \, /{\text{ N}}$$

where Yn = yield in treatment with N applied, Yo = yield in treatment without N applied, N = unit N applied.

### Statistical analysis

Significant differences of the mean were statistically analyzed with ANOVA and the differences were reported throughout are significant at *p* ≤ 0.05 using SAS software package 9.2. All diagrams were drawn using GraphPad Prism 9 v9.4.1 software (2022).

### Ethics approval and consent to participate

This manuscript is an original paper and has not been published in other journals. The authors agreed to keep the copyright rule.

## Results and discussion

### Biometric observation

**Figure 1:**
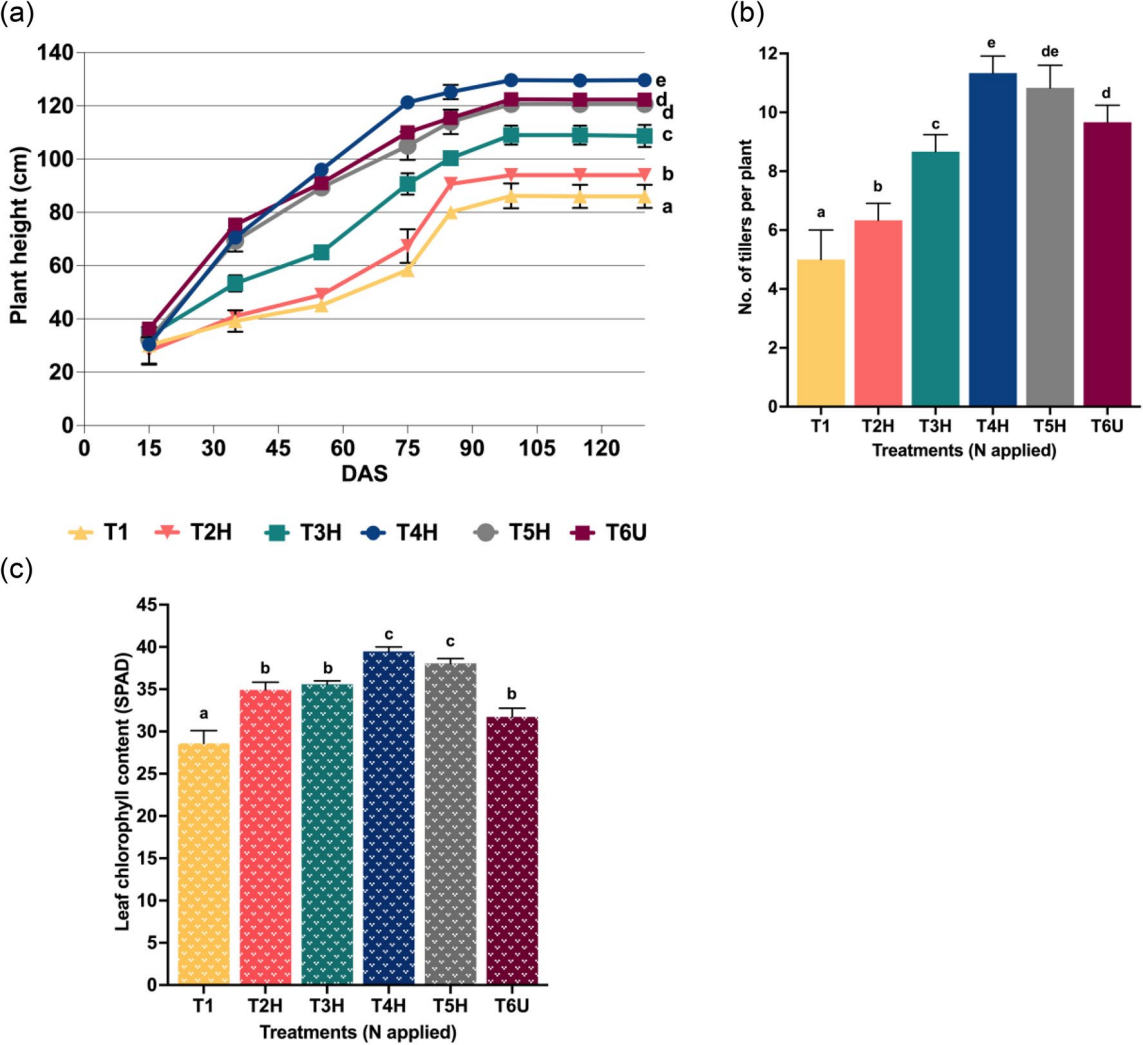
Effect of N content on the (**a**) plant height (cm), (**b**) number of tillers and (**c**) leaf chlorophyll content (SPAD) of rice plants. Error bars show the standard deviation from 5 replications (n = 5). Different letters indicate significant differences (*p* ≤ 0.05).

Plant height increased with all treatments with time until it reached the maturity stage at 130 DAS (Fig. 1a). The highest rice plant height (129.6 cm) was observed in treatment T4H (4.8 N g pot^−1^ = 75% RDF) whereas the lowest plant height (86.1 cm) was observed in T1 (0 N). Plant height using T5H (120.7 cm) was similar to the treatment given common urea dosage (T6U, 122.4 cm). Other treatments recorded plant heights of 109.4 cm (T3H) and 94.1 cm (T2H). The data demonstrates that the growth of the plants in T4H initially delayed behind those given common urea, T6U and higher dose of N in T5H. However, from 30 to 45 DAS, the plants were almost of the same height and after this point, the plants in T4H outperformed the plants in T6U and T5H. Also, T4H was able to maintain the plant height and T5H did not have the drastic improvement in the plant height. These observations show that CRF with 75% N (T4H) has a more sustained release of fertilizer in soil than common urea. Generally, N increases the protein content in plants^12, 33, 34^. When the plants receive a sufficient supply of N, protein is available at an optimal level to accomplish metabolic processes, which improves the vegetative and reproductive growth of the plant and the plant yield^35–37^. Improved growth is associated with higher plant height. Similar trends with rice have been observed in other studies using encapsulated urea which shows higher plant height than rice treated with conventional urea^15, 27, 38^.

Application of 75% N in T4H produced the most tillers per plant (12), followed by T5H (11) and T6U (9) (Fig. 1b). Higher number of tillers in CRF confirmed the findings from another study which demonstrated that the administration of CRF may increase the number of tillers in rice compared to that of common urea^15, 39^. This is most likely due to sustained release and sufficient N available to the rice plants^15, 39^.

Measurement of leaf chlorophyll content is a crucial metric to assess general plant health and for improving NUE^40^. It suggests the physiological status of plants which are attributed to the photosynthetic pigment and leaf N content^41^. Also, the chlorophyll level of leaf tissue is significantly affected by N availability in soil^41, 42^. In this study, maximum SPAD reading was 39.5 (T4H) and followed the order of T4H > T5H > T3H > T2H > T6U > T1 (Fig. 1c). CRF at 75% (T4H) and 100% (T5H) led to a modest improvement in the leaf photosynthetic rate. This demonstrates that SPAD readings were associated and linearly correlated to the chlorophyll and N contents in the plants^43, 44^. A higher SPAD reading indicates a higher chlorophyll content as a result of better N uptake. Increased N uptake by the plant increases the protein content in the plant^33^, which promotes higher photosynthetic rates thus improving the plant growth^36^. Dobermann and Fairhurst^45^ suggested that the optimum SPAD threshold for rice is between 29 and 32. In this study, T4H (39.5) and T5H (38.1) raised the SPAD readings for rice plants surpassing the optimum SPAD threshold. Meanwhile, T6U treatment with common urea gave a SPAD reading of 31.7 which still falls within the optimal threshold range. Increased SPAD readings indicate that CRF in T4H and T5H administration increased N contents in the rice plants as a result of better N uptake by plants while minimizing N loss to the environment^35^. Similar results have been reported in other studies^46, 50^.

In addition, application of 75% N in T4H performed better in increasing growth performance of rice compared to other CRFs as reported in other studies, for instance, urea super granule, USG (plant height: 115 cm, 15 tillers at 104 kg N ha^−1^)^21^, palm stearin coated urea, PS + DMPP-50 (plant height: 113.2 cm, SPAD: 37.4 at 50.4 kg N ha^−1^)^15^, biochar impregnated urea, BIU 700–5 (plant height: 116.9 cm, SPAD: 34.35 at 52.8 kg N ha^−1^)^15^.

### Yield and yield component data

**Figure 2:**
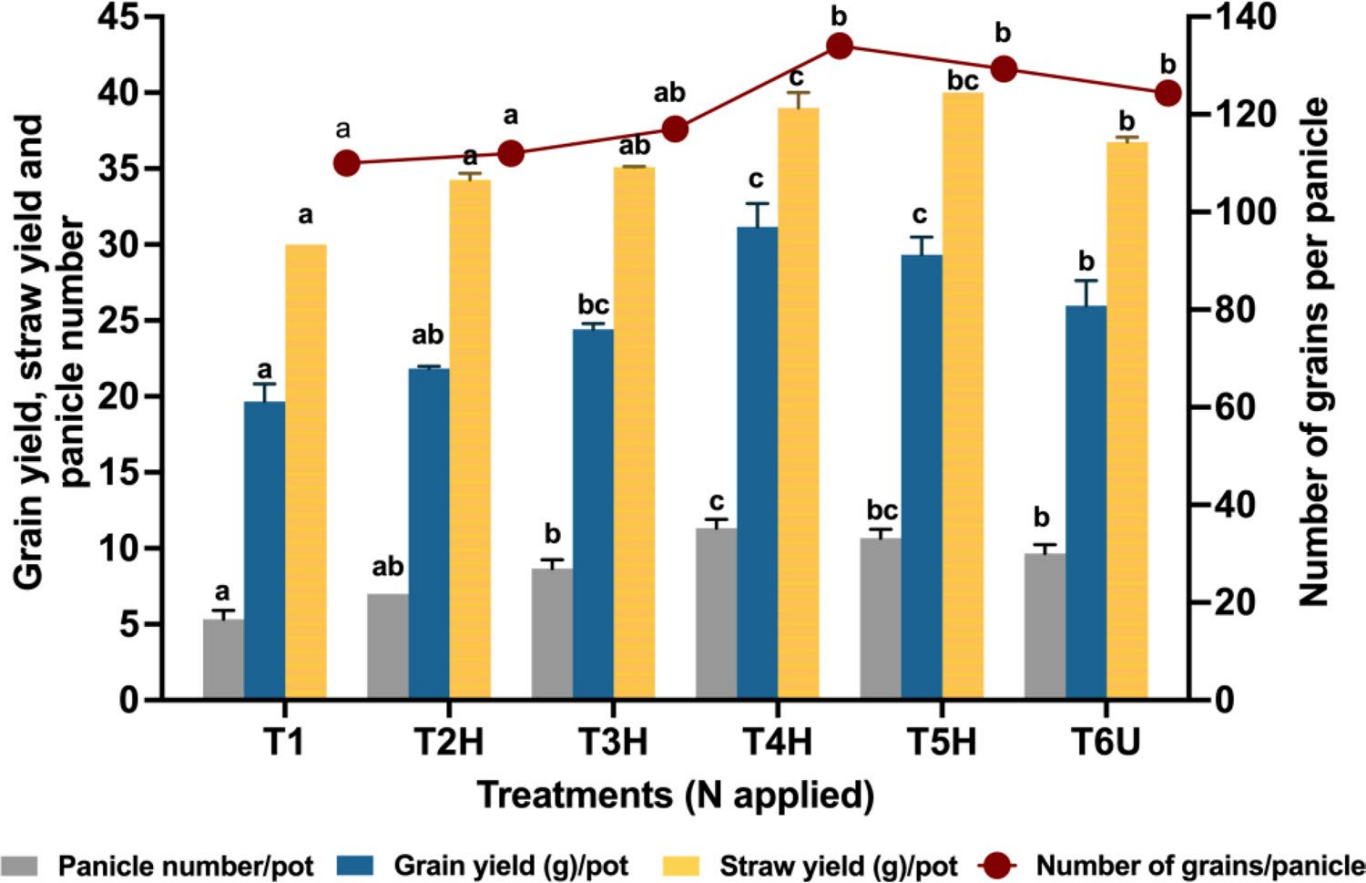
Effect of N treatments on the number of panicles per plant, number of grains per panicle, grain and straw yield. Error bars show the standard deviation from 5 replications (n = 5). Different letters indicate significant differences (*p* ≤ 0.05).

The N treatments had a substantial effect on the yield and yield parameters of rice plants (Table 2 and Fig. 2). Yield components included 1000-grain weight, percentage filled grains (PFG), panicle number and number of grains per panicle. T4H showed the highest 1000-grain weight (24.85 g) albeit not significantly different from other treatments (T3H, T5H and T6U) (Table 2). Previous studies reported that N management had no substantial effects on 1000-grain weight^51, 53^. In addition, further increase in N might reduce1000-grain weight, most likely due to insufficient carbohydrates to fill the larger number of grains produced. PFG varied with N treatments where PFG was the highest in T4H, followed by T5H and T6U, with the lowest in T1.

**Table 2:** 1000-grain weight (g) and percentage filled grain (%F) of different N treatments.

Treatments	1000-grain weight (g)	Percentage filled grain (%F)
T1	20.46^a^	71.74^a^
T2H	20.52^a^	73.36^a^
T3H	21.76^ab^	77.69^b^
T4H	24.85^b^	89.59^c^
T5H	24.06^b^	87.9^c^
T6U	23.25^b^	72.38^a^

Different letters indicate significant differences (*p* ≤ 0.05).

The number of panicles in each pot ranged from 6 to 12 panicles pot^−1^ (Fig. 2). T4H produced the most panicles (12 panicles pot^−1^), followed by T5H (10 panicles pot^−1^). Rice plants treated with common urea, T6U, and T3H (CRF 50% N) produced the same panicles number (8 panicles pot^−1^) and T1 produced the fewest panicles (6 panicles pot^−1^), as reported in previous research which found increased panicle number with increased N rates^54, 55^. Also, higher N rates might indicate more N availability during the tillering stage, which is important for cell division^56^. Cell division induced by N supply promotes panicle formation during reproductive phases of the rice crops^57^. Highest number of grains per panicle was observed in T4H (134) followed by T5H (128) and T6U (123). T1, the treatment with no N fertilizers, produced fewer grains (110). The higher number of grains with increasing N rates could be attributed to a high number of panicles, resulting in high grain yield in rice^58, 59^.

Significant increases in grain yield were recorded by T3H, T4H, T5H and T6U (Fig. 2). In terms of percentage, T4H increased grain yield by 71.0% when compared to the control treatment, T1. The increase in grain yield (%) of T4H was also greater than in other treatments, with T2H recorded 15% increase, T3H 33% increase, T5H 58% increase, and T6U 46% increase. In this study, CRF with 75% N (T4H) administration in soil improved the grain yield when compared to the CRF with 100% N (T5H) and 100% RDF (T6U). This suggested that CRF application to soil could minimize N fertilizer consumption while enhancing crop production^48, 60^. Also, these results were consistent with other studies^61, 62^, suggesting that a moderate N application, i.e., T4H in this study, might show the highest yield potential, and further increase in N supply did not increase grain yield. Moreover, this shows that CRF might deliver a sustained N supply to rice plants via sustained N release, which is vital to increase N uptake at late growth stages of rice, thus increasing grain yields^49, 50^. This was further supported by Zhang et al.^54^ who discovered that increasing N rates significantly increased grain yields; however, this increase in grain yield is limited to a certain range of N rates. The positive effects of CRF on the yield and yield-attributing factors compared to that of T6U (common urea) were consistent with the results of previous studies^15, 61, 62^.

Relative to CRF treatment in this study (T4H), early reported CRFs showed lower percent increase of grain yields; USG (54.35% at 104 kg N ha^−1^)^21^, PS + DMPP-50 (13.63% at 50.4 kg N ha^−1^), BIU 700–5 (13.10% at 52.8 kg N ha^−1^)^15^, resin-coated urea, RCU (35.6% at 216 kg N ha^−1^) and polyurethane-coated urea, PCU (30.3% at 216 kg N ha^−1^)^63^. Moreover, T4H produced higher grains per panicle (134) compared to other CRFs; USG (124), RCU (116) and PCU (122). However, T4H showed no significant different for 1000-grain weight; USG (22.65 g), BIU 700–5 (26.39 g), PS + DMPP-50 (26.37 g), RCU (24.5 g) and PCU (24.8 g). Findings from this study showed that CRF treatment of T4H performed better in increasing grain yield and yield components of rice compared to other CRFs.

#### Harvest index (HI)

**Figure 3:**
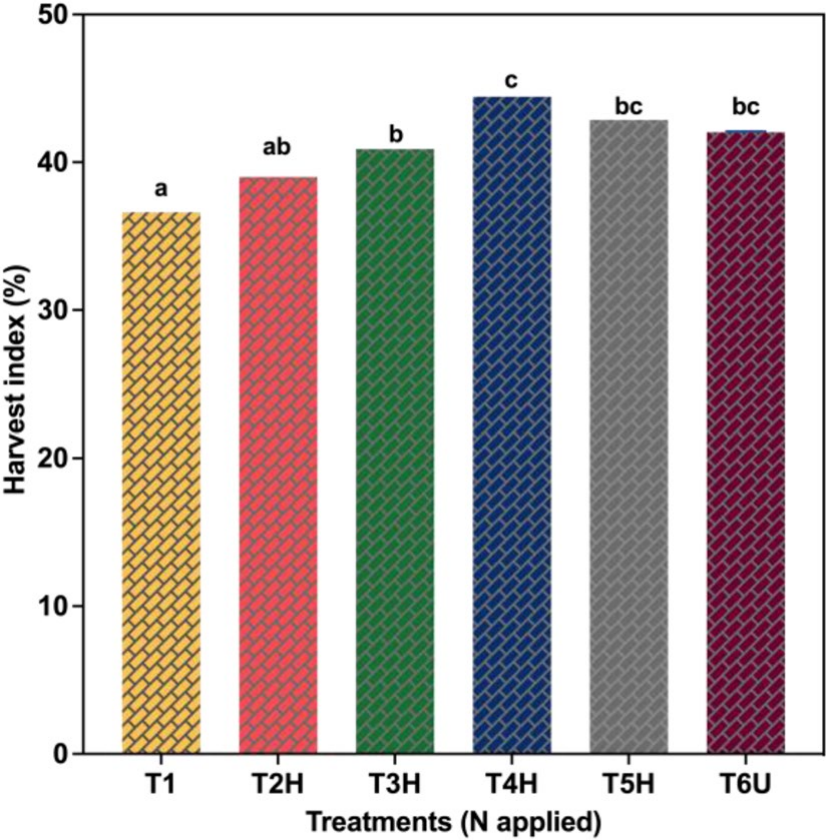
Harvest index of rice with different N treatments. Different letters indicate significant differences (*p* ≤ 0.05).

Higher HI indicates that more of the harvested aboveground parts of rice were grains^64^. Therefore, higher HI is preferable since the grain yield is the most important aspect of rice cultivation. Figure 3 shows that all CRF treatments improved rice HI with T4H recorded the highest HI (45.5%). T5H showed slight improvement in HI albeit not significantly different than that of T4H and other treatments. The observed increase in HI for T4H could be attributed to an increase in total N uptake in the rice plants that peaked during the 11th week of grain formation^65, 66^. After the 11th week, during the grain filling and maturation stage, most of the N required by rice comes from the culm, leaves and panicles rather than the soil^65, 67^. This suggests that N uptake from the soil was minimal^67^. This is most likely a primary factor that improves HI. More N is likely be transported to the grains as the plants absorbed sufficient N before the 11th week, enhancing HI and grain yield over biomass.

#### Dry matter partitioning

**Figure 4:**
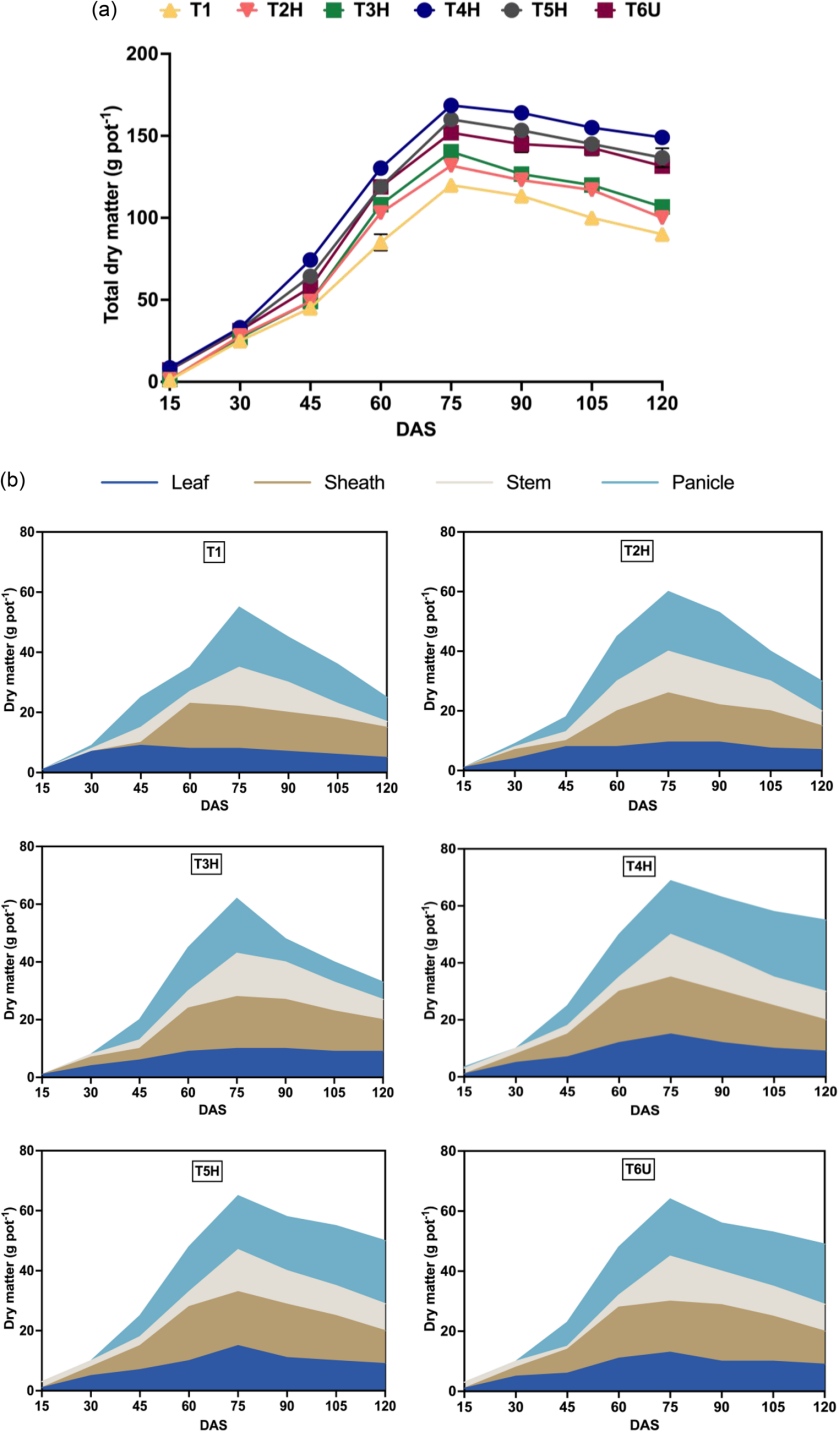
(**a**) Dry matter accumulation in rice as influenced by N rates application over the crop growth stages. (**b**) Pattern of dry matter partitioning to different parts of rice as influenced by time course and nitrogen levels.

Dry matter progression in the rice plants increased steadily throughout the growth phases and peaked at maturity (Fig. 4a). The dry accumulation pattern in the rice plants demonstrated a modest exponential growth, period of linear growth, and a period of constant weight. The highest dry matter (109.9 g pot^−1^) was obtained in T4H, while the lowest (62.3 g pot^−1^) dry matter was obtained in control treatment, T1. Dry matter in T5H and T6U was 107.9 g pot^−1^ and 107.1 g pot^−1^, respectively. Results thus indicate that response was more pronounced with higher N rate applications than lower N rate applications.

Higher N rates can increase dry matter content due to higher protein content in the rice plants, which promotes higher photosynthetic rates on leaves^33, 34^. This enhanced the rice plants growth during vegetative stage and later distribution of assimilates to the reproductive organs^33, 34^. Furthermore, dry matter production is profoundly associated with intercept photosynthetically active radiation^43^. Low N supply in have been identified as a limiting factor in alleviating radiation usage efficiency and biomass productivity, resulting in lower dry matter yield [43,444]. Information on dry matter production and partitioning between different plant sections as influenced by N rates application is crucial to develop rice growth models^33, 34, 43, 44^. Dry matter partitioning to the reproductive organs depends on number, capacity, and activity of physiological sinks^44^.

The distribution of dry matter into leaf, leaf sheath, stem and panicles of the rice plants was nearly identical across the treatment levels (Fig. 4b). The portioning of dry matter into leaf, leaf sheath, and stem increased up to 65 DAS, while panicle dry weight rose until 75 DAS. After 65 DAS, dry matter partitioning into vegetative parts reduced, indicating remobilization of assimilates from vegetative parts towards flowering stages and forming grains^43^. N fertilization induced a considerable variation in the pattern of dry matter partitioning of the rice plants at all growth stages, where low N rates stimulate allocation of least dry matter to all plant sections.

### Plant nitrogen uptake and efficiencies

**Figure 5:**
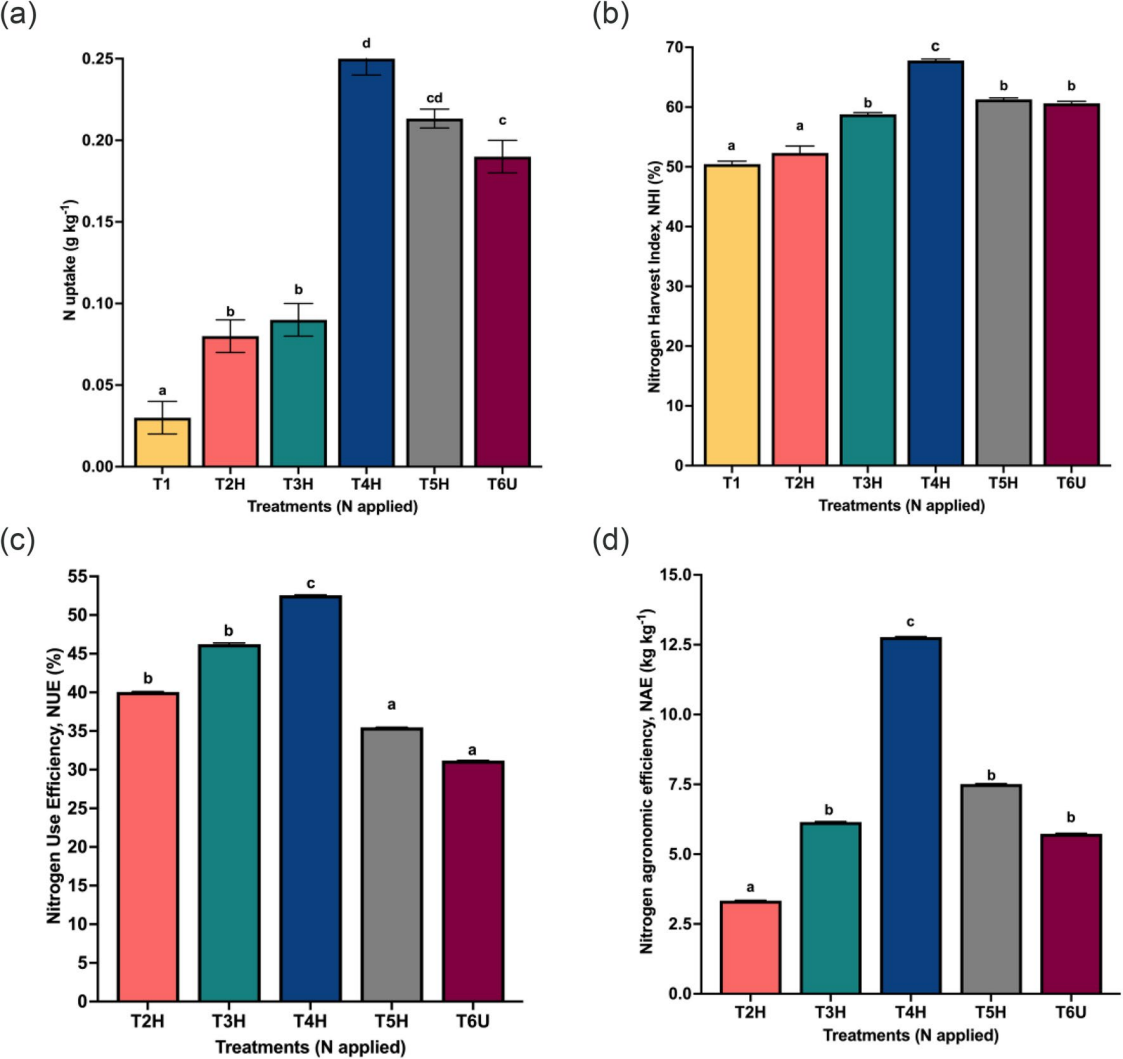
Nitrogen uptake and efficiencies by different N treatments. (**a**) N uptake, (**b**) nitrogen harvest index (NHI), (**c**) nitrogen use efficiency (NUE) and (**d**) agronomic nitrogen efficiency (NAE). Error bars show the standard deviation from 5 replications (n = 5). Different letters indicate significant differences (*p* ≤ 0.05).

Figure 5 shows the influence of N treatments on the rice N uptake and N use efficiencies throughout the experiments. Figure 5a shows the optimum N uptake rate in the presence of CRF hydrogels with the highest N uptake observed in T4H in this study. The rice N uptake in different treatments showed significant differences at p value less than or equal to 0.05. The N uptake of crop ranged from 0.03 g kg^−1^ (T1, lowest) to 0.25 g kg^−1^ (T4H, highest) with T5H recording the second highest N uptake in rice crops (0.22 g kg^−1^). CRF applications in T4H and T5H significantly improved the rice N uptake compared to those treated with conventional urea (T6U, 0.19 g kg^−1^) at higher N applied. However, crop N uptake was relatively lower in T2H (0.08 g kg^−1^) and T3H (0.09 g kg^−1^). The trend of rice N uptake is consistent with findings by Hashim et al.^15^. Increases in N uptake is closely associated to increased aboveground yield (Fig. 2) because the crop N uptake exhibited a trend like that of plant biomass.

Nong et al.^68^ reported that an increase in N uptake with CRF application could be due to N translocation from vegetative parts of the rice plants to grain during the later stages of paddy plant growth. Further, urea-loaded hydrogels were observed to facilitate the gradual release of nutrient that closely matched the N requirement of the rice crop, thus improved N use efficiencies. These results are similar to those reported by Fazlina et al.^38^. T4H had the highest NHI (67.9%) of all N treatments (Fig. 4b). The second highest NHI was observed in T5H (61.3%), followed by T6U (60.5%). The NHI values for T2H, T3H and T1 were 53.0%, 58.9% and 50.4%, respectively. Based on our finding, the highest NHI in T4H indicates positive synchronization between increased grain yield (Fig. 2) and HI (Fig. 3). Other treatments also showed positive synchronization with similar NHI patterns as T4H.

In Fig. 4c, the highest NUE (52.6%) was observed in T4H, while the lowest NUE (31.2%) was computed by treatment T6U. Generally, the NUE of CRF treatment increased as a function of N application rate up to 4.8 N g pot^−1^ (T4H), after which NUE began to decrease with increasing N rate (6.4 N g pot^−1^ in T5H and 6.4 N g pot^−1^ in T6U). This is consistent with that of previous studies indicating that moderate N rate might lead to better NUE potential because further increase in N rate does not increase the NUE values^62, 63^. Also, studies have found that conventional urea in farmers’ practices have low NUE^69^, resulting in poor synchronization between the N supply and crop demand. This was validated in our current study, where T6U showed the lowest NUE of all N treatments.

NAE ranged from 3.33 to 12.78 kg kg^−1^ for different N rates, with T4H having the highest NAE (12.78 kg kg^−1^) in Fig. 4d. NAE rose with increasing N rate for CRF up to 4.8 N g pot^−1^ in T4H (90 kg N ha^−1^), but after that, declining NAE with increasing N rates was observed in T5H and T6U. The NAE values in T5H and T6U were still higher than in T2H and T3H due to higher grain yields in T5H and T6U (Fig. 2). Barbieri et al.^70^ reported similar results, with NAE decreasing as the N application rates increased.

One of the primary objectives of N fertilization is to enhance N uptake and N efficiencies in upland rice systems using cellulose-based hydrogels as controlled release fertilizers. In our study, the highest N efficiencies were observed in rice plants treated with CRF at 4.8 g N pot^−1^ (T4H) compared to the traditional farmers’ fertilizer practices using common urea. This could be explained by the level of N rate in T4H corresponding with the optimal crop N requirement. Collectively, our results are consistent with data from several studies as reported by Wang et al.^56^, Abbasi et al.^32^, Barbieri et al.^70^ and Yi et al.^71^.

Moreover, T4H showed significantly higher N uptake compared to other CRFs reported in other studies, i.e., USG (0.14 g kg^−1^), PS + DMPP-50 (0.20 g kg^−1^), BIU 700–5 (0.21 g kg^−1^), RCU (0.16 g kg^−1^) and PCU (0.17 g kg^−1^). Other CRFs also have lower NAE when compared to T4H in this study such as RCU (7.5 kg kg^−1^) and PCU (9.4 kg kg^−1^). This shows that T4H has better performance in enhancing N uptake and efficiencies than other CRFs.

### Correlation regression analysis

**Figure 6:**
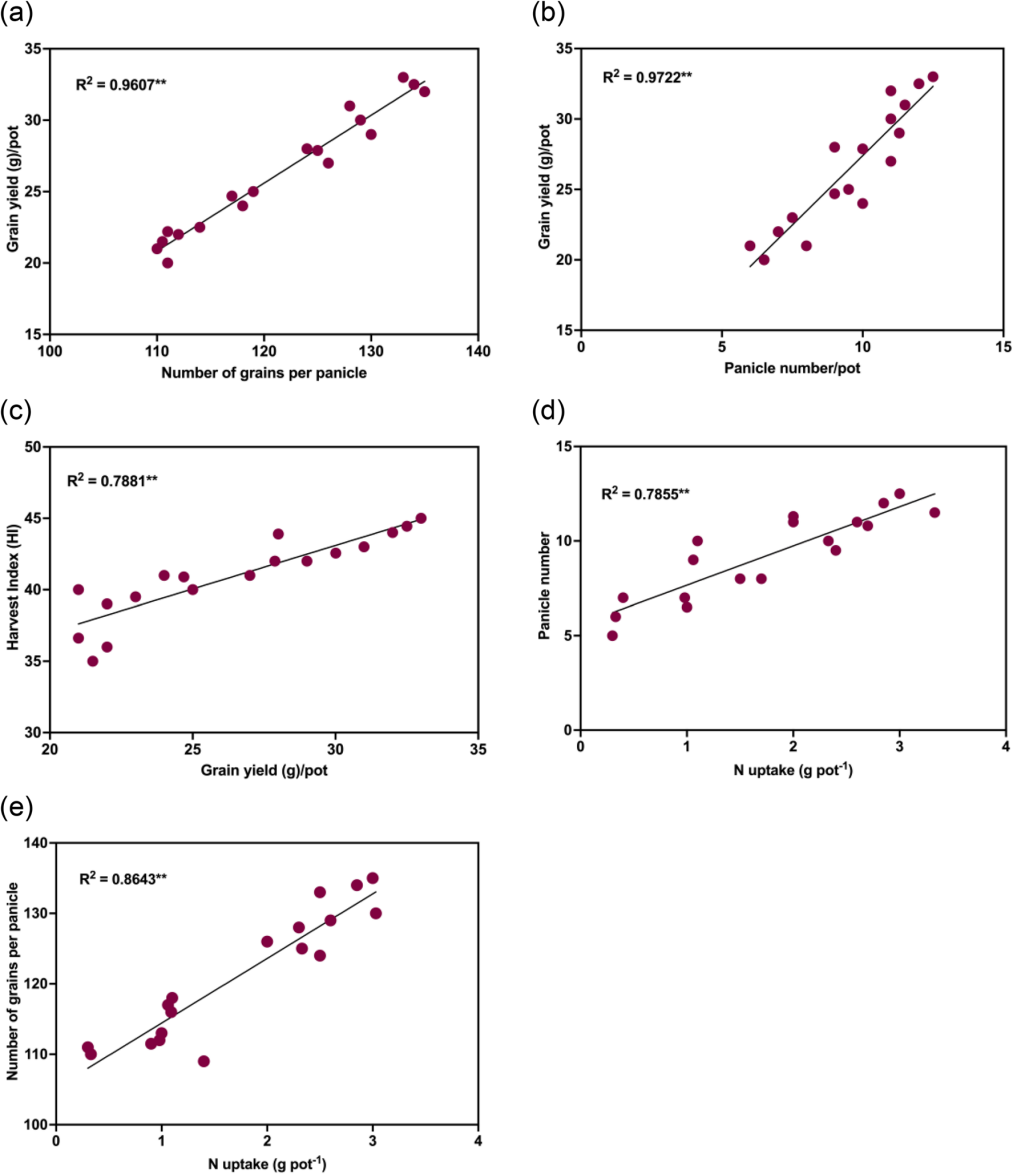
Linear relationship of grain yield with (**a**) number of grains per panicle, (**b**) panicle number/pot, (**c**) harvest index (HI) and linear relationship of N uptake with (**e**) panicle number and (**f**) number of grains per panicle at *p* < 0.05**

Sui et al.^72^ reported that the increase in grain yield was primarily due to increased panicle number and number of grains per panicle which agrees with our findings that the panicle number and number of grains per panicle were the most variable yield components identified, i.e., T4H. Considering the N fertilization, the highest grain yield for T4H could be attributed by the increased panicle number and number of grains. This was further corroborated by the significant and positive correlation between grain yield and both the number of grains per panicle (*R*^2^ = 0.9607, *p* < 0.05) and panicle number (*R*^2^ = 0.9722, *p* < 0.05), as shown in Figs. 6a and b. Panicle number is one of the contributing factors determining yield. This is because increasing panicle number has a similar effect to increasing grain yield^57, 73^. Furthermore, current results showed that panicle number and the number of grains per panicle were both positively correlated with N uptake (Fig. 6d, e). This shows that increased N uptake enhanced grain formation, resulting in higher grain yield. In addition, grain yield thus accounted for 78.8% of the increase in HI, whilst variables other than HI contributed the remaining 21.2% of the increase in grain yield (Fig. 6c).

CRF treatments have significantly increased rice yields, which was also observed in present study, especially in T4H. A possible explanation for this might be due to N supply from the CRF closely met the N requirements of the rice crop. Urea fertilizers encapsulated in cellulose hydrogels were observed to facilitate gradual N release during the growing cycle, allowing rice plants to absorb sufficient N efficiently, while minimizing N losses via leaching and ammonium volatilization^74^. A single basal application of CRF significantly reduced N leaching compared to split application of conventional urea by farmers’ fertilizer practices, with no loss of crop yield^13^. Yang et al.^50^ and Ni et al.^75^ also found that CRF provided controlled N release over time to better match crop N requirements throughout the growing season. Hence, CRF offers an attractive alternative to mitigate environmental N losses in rice production by enhancing N efficiencies compared to conventional urea^76^. Cao et al.^77^ suggested that increased N efficiencies in rice was due to increased N assimilatory activity. Further, an important element of N assimilatory enzyme, glutamine synthetase activity is positively correlated with the N utilization of rice crops^36, 78^. These findings further support the results from this study that CRF treatment, i.e., urea-loaded cellulose-based hydrogels increased grain yield and enhanced N efficiencies.

It is noted that although T2H and T3H involved CRF utilization, both treatments did not give substantially higher yields unlike T4H possibly due to insufficient N application rate to meet the rice crop’s optimal requirement. Another possible reason contributing to the excellent performance of T4H the urea-loaded cellulose hydrogels (CRF) was remained longer in soil at high enough N content and existed in the N form available for rice plants could absorb throughout the cultivation period. This could potentially be associated with the rate of N in the soil before and after rice cultivation. In this study, the rate of N release in the soil before rice cultivation was 75%^18^ and the rate of N release after rice cultivation was 87%. The remaining 13% N existed in CRF, maintained in the soil and degraded over time. After rice cultivation, small fragments of CRF hydrogels were retrieved, and the CRF weight loss was determined (up to 97%) compared to their initial weight. This shows that CRF hydrogels have nearly 100% degraded after 135 days. Thus, CRF with this ability may offer many advantages for rice cultivations on the hill, including reduced N losses and frequent dosing, thus enhancing plant growth and quality to give higher yields, greater N efficiencies^13^. Furthermore, CRF can be applied as a single basal dose, making it convenient for farmers to implement in their farming practices, owing to less frequent dosing and thus, lower labor cost.

## Conclusion

The results of present study showed that CRF with 75% N applied (T4H; 4.8 g N pot^−1^ = 90 kg N ha^−1^) with a single basal application could produce higher rice grain yields and N efficiencies compared to those resulting from split applications of urea at 100% N RDF. Relative to control, CRF hydrogels (T4H) increased rice yield by 71% with the highest NUE (52.6%) and NAE (12.8 kg kg^−1^). The yield component analysis revealed that, the higher rice grain yields from the CRF treatment (T4H) were associated to an increased panicle number and number of grains per panicle in response to the CRF treatment. Also, further increase in the N rate applications did not further increase the rice grain yields and N efficiencies (T5H and T6U). In addition, in most cases, there are significant differences in the N uptake of rice crops among the CRF and urea treatments. This study suggested that practically, farmers should be advised to apply CRF hydrogels at 75% N rates for substantial rice production. The findings in this study highlight the potential of urea-loaded cellulose hydrogel as CRF as an enhanced agronomic alternative for upland rice cultivation, increasing input efficiencies and rice productivity.

## Data Availability

The data sets used and/or analyzed during the current study are available from the corresponding author on reasonable request.
